# The blackcap (*Sylvia atricapilla*) genome reveals a recent accumulation of LTR retrotransposons

**DOI:** 10.1038/s41598-023-43090-1

**Published:** 2023-09-30

**Authors:** Andrea Bours, Peter Pruisscher, Karen Bascón-Cardozo, Linda Odenthal-Hesse, Miriam Liedvogel

**Affiliations:** 1 MPRG Behavioural Genomics, Max Planck Institute for Evolutionary Biology, 24306 Plön, Germany; 2 Department of Evolutionary Biology, Evolutionary Biology Centre (EBC), Uppsala University, Uppsala, Sweden; 3 Department Evolutionary Genetics, Max Planck Institute for Evolutionary Biology, 24306 Plön, Germany; 4 Institute of Avian Research “Vogelwarte Helgoland”, 26386 Wilhelmshaven, Germany

**Keywords:** Evolutionary genetics, Molecular evolution, Genome, Genomics

## Abstract

Transposable elements (TEs) are mobile genetic elements that can move around the genome, and as such are a source of genomic variability. Based on their characteristics we can annotate TEs within the host genome and classify them into specific TE types and families. The increasing number of available high-quality genome references in recent years provides an excellent resource that will enhance the understanding of the role of recently active TEs on genetic variation and phenotypic evolution. Here we showcase the use of a high-quality TE annotation to understand the distinct effect of recent and ancient TE insertions on the evolution of genomic variation, within our study species the Eurasian blackcap (*Sylvia atricapilla*). We investigate how these distinct TE categories are distributed along the genome and evaluate how their coverage across the genome is correlated with four genomic features: recombination rate, gene coverage, CpG island coverage and GC content. We found within the recent TE insertions an accumulation of LTRs previously not seen in birds. While the coverage of recent TE insertions was negatively correlated with both GC content and recombination rate, the correlation with recombination rate disappeared and turned positive for GC content when considering ancient TE insertions.

## Introduction

Transposable elements (TEs) are classes of repetitive genetic elements with the ability to move across the genome. They most commonly reside within the non-coding part of the genome. TEs can move around the genome by either copy-pasting themselves (Class I elements or retrotransposons) or behaving in a cut-and-paste manner (Class II elements or DNA transposons). These two classes are further subdivided into orders defined by their respective repeat sequence and transposition characteristics^1^. While typically both classes are found within most species, their abundance differs considerably between organisms. Avian genomes, for example, are known to have a low proportion of TEs in their genome which show a reduced overall TE diversity, with the biggest proportion attributed to the chicken repeat 1 (CR1) superfamily of long interspersed nuclear elements (LINEs) and the second largest to long terminal repeat transposons (LTRs)^2^. Through their ability to move around and accumulate, TEs can have a profound evolutionary impact on their host’s genomes.

The effect of a TE on its host can be classified analogous to the effect of point mutations. In the majority of cases, the consequences of a TE their activity (transposition to a new genomic site) is either neutral or deleterious. The latter occurs, when TEs disrupt genes and their functions, or when, they trigger *de-novo* genomic instability by transposition or TE-mediated chromosomal rearrangements, which can lead to disease^1, 3^. TEs can occasionally have a positive impact on the host genome, for example, by impacting gene regulatory networks. In the British peppered moth (*Biston betularia*), a TE inserted within the first intron of the cortex gene, resulted in increased transcription levels, subsequently affecting cell cycle regulation during wing-disc development through the amount of cortex protein product, resulting in the iconic melanic form^4^. However, more research is needed to understand these different evolutionary impacts that TEs can have when interacting with their host genome.

The increased accessibility to high throughput sequencing technologies has greatly increased our ability to analyse genetic differences caused by changes at the nucleotide level, and patterns of natural selection on coding sequences, and simultaneously allowed us to disentangle phenotypic differences at the nucleotide level. Mounting evidence has started to shed light on non-coding regions having important effects on genomic variation^3^. While TEs can be found in the genomes of virtually all organisms, large proportions of TEs are often absent from reference genomes, as their repetitive nature impedes their assembly and can result in collapsed regions within the reference genome^2, 5^. These difficulties have led to an increased demand for reference genomes that are of a higher quality and are more complete. More importantly, a new demand for high-quality annotations of non-coding regions in reference genomes has surfaced. Annotations of non-coding regions are imperative to study the evolution of these regions between and within species. Improvements in sequencing techniques, especially the addition of long-read sequencing, and improved bioinformatic analytical tools are resulting in the assembly of increasingly gapless reference genomes, enabling the curation of high-quality TE annotations.

The current efforts of large consortia, such as the VGP^6^ and the B10K^7^ to create high-quality references for a wide variety of organisms provide invaluable data to improve our endeavours for a better understanding of TEs. With these new resources we can take our research into TEs and their effects on host genomes further, for example, to better understand the evolution of complex traits across phylogenomic scales. One such a complex trait is seasonal bird migration and recent research across a migratory divide in willow warblers identified a diagnostic TE correlated with migratory direction^8^. Here we focus on the Eurasian blackcap (*Sylvia atricapilla*), another iconic model species for bird migration, and consequently, the resource published here may be able to add insight to the quest to resolve the genetic background of migratory behaviour.

Here we present a high-quality TE annotation across the Eurasian blackcap genome^9, 10^, and the TEs relation to specific genomic features, i.e. chromosome length, gene coverage, recombination rate, GC content and CpG islands. We used an approach to analyse TEs distinctly for recent and ancient TE insertions, further advancing the study of TEs and their effect on the genome as well as on phenotypic traits. Our approach leverages the information from the kimura-2 distance parameter, which is typically calculated when annotating a genome’s TEs. This serves as a complement to TE annotation studies when (manually curated) TE annotations of closely related species are not available. This approach offers a first look into recent TE insertions compared to ancient insertions within the blackcap genome. We hope that the TE annotation as presented here provides a useful resource to the research community to further investigate evolutionary processes that are involved in, for example, complex traits, as well as providing a blueprint that may inspire similar analyses for other high-quality reference genomes across a wide range of taxa.

## Results

**Figure 1:**
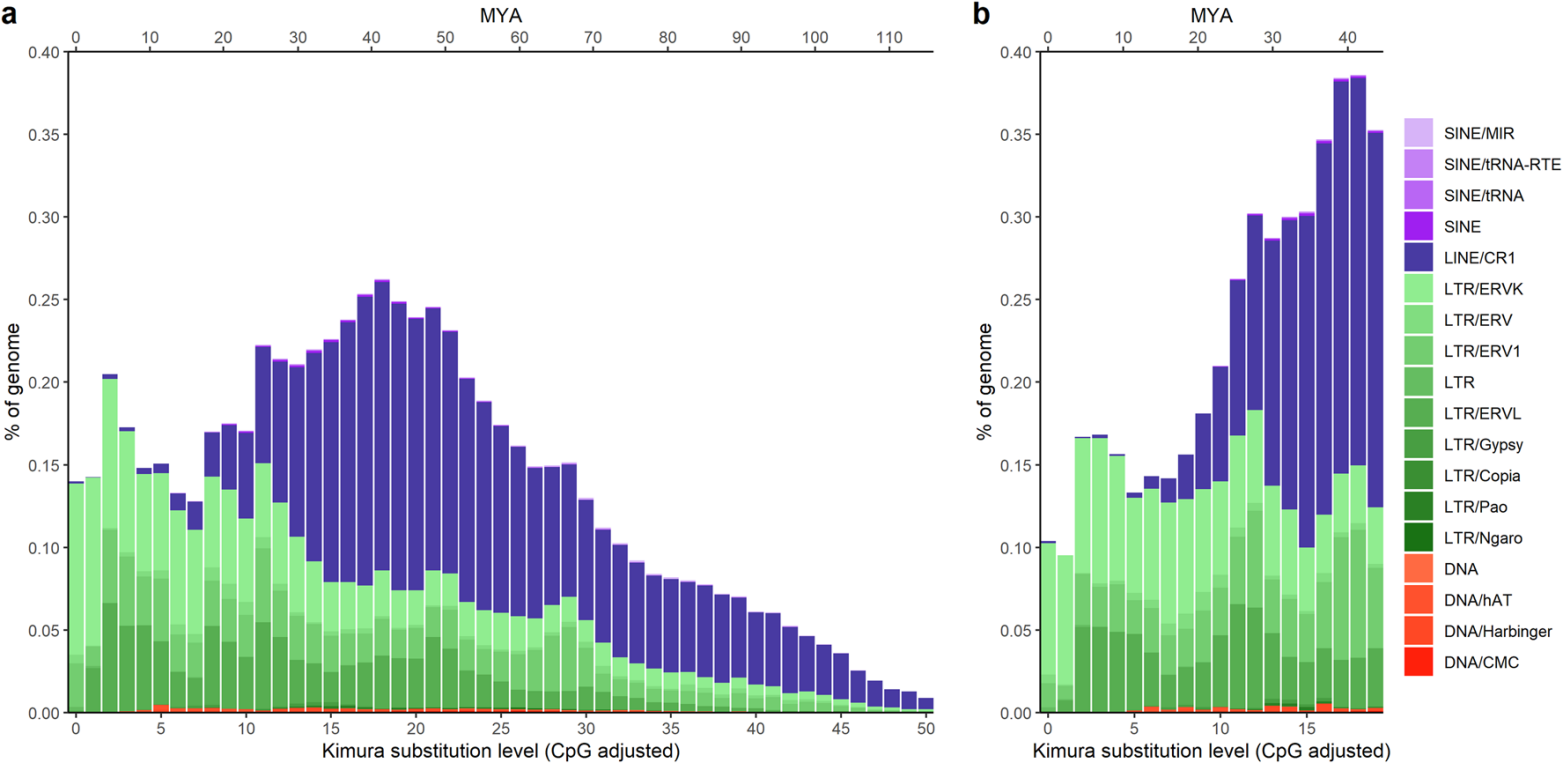
Interspersed repeat landscape of the blackcap genome. (**a**) Repeat landscape calculated by RepeatMasker on the raw output. (**b**) Repeat landscape calculated on the final curated TE annotation (see Materials and Methods for more details). The bottom x-axis shows the kimura-2 substitution level (CpG adjusted), the top x-axis is the timescale in million years ago (MYA), and the y-axis is the percentage of the genome occupied. Colour coding of the different repeat types/families found is listed to the left.

We present a high-confidence annotation repeat landscape for the blackcap genome, generated by combining thoroughly filtered de novo predictions of repeats and manually curated libraries of bird TEs (see materials and methods for more details). Through our RepeatMasker run, we classified a total of 7.68% of the genome as interspersed with TEs (Table 1), dominated by LTR and LINE elements, covering ~ 54% and ~ 43% of the total repeat content, respectively. In contrast, short interspersed elements (SINEs) and DNA elements only accounted for ~ 2% (~ 0.5% and ~ 1.5% respectively) of all TEs annotated in the blackcap genome. In contrast, our final TE annotation, for which we combined copy fragments of TEs according to the 80–80–80 rule^11^, contains only the (merged) TE copies with a minimum base length of 80, at a minimum of 80% similarity to the reference sequence of the element and has a minimum of 80% identity to the reference sequence of the host. The merging of Repeatmasker TEs according to the 80–80–80 rule results in decreasing substantially both the total number of TE copies found and their coverage along the genome (Table 1), while the identity threshold resulted in TE copies with a kimura-2 parameter of 20 and more to be filtered out. Our final TE annotation covers a total of 5.06% of the reference genome, of these ~ 63% are LTRs and ~ 36% are LINEs, with SINE and DNA elements comprising ~ 1%. We estimated the relative distance of each TE to their consensus sequence using the Kimura-2 parameter distance to each TE copy, for both the raw RepeatMasker output as well as the final TE annotation. Furthermore, we calculated an approximate age in millions of years of the Kimura-2 parameter distribution using the estimated mutation rate of the collared flycatcher. This revealed TE landscapes with a recent expansion of LTR elements, as well as more ancient LINE expansion and reduction (Fig. 1). The recent expansion of LTR elements, specifically Endogenous retrovirus K-promotor (ERVK) elements (Supplementary Fig. S1), is visible by the elevated levels of genome coverage of LTRs at low substitution levels (< = 2), which thus appear currently active at a high level (Fig. 1). This is supported by the LTR elements making up more than 60% of the TE genome-wide coverage, while only accounting for ~ 41% of the total TE copies found within the genome. In comparison, the reduction of the LINE expansion is visible through the decline in coverage, with decreasing kimura substitution levels (Fig. 1) and a higher amount of copy fragments as shown in Table 1. From here on out we use the final TE annotation to perform our analyses.

**Table 1:** Summary of RepeatMasker annotation and final TE annotation. Showing repeat type, copy or fragment number, total occupied length in base pair (bp) and percentage of the genome assembly covered by each repeat type, for both the raw RepeatMasker annotation and the final TE annotation presented.

Repeat type	RepeatMasker			Final TE annotation		
	Copies	Total length (bp)	% of genome	Copies	Total length (bp)	% of genome
SINE	4269	463,447	0.04	1466	182,635	0.02
LINE	127,084	35,233,072	3.34	61,906	19,307,025	1.83
LTR	82,330	43,924,839	4.16	45,188	33,395,659	3.17
DNA	4279	1,160,803	0.11	965	483,169	0.05
Unclassified	487	204,826	0.02	–	–	–
Total interspersed repeats		80,986,987	7.68		53,368,488	5.06

**Figure 2:**
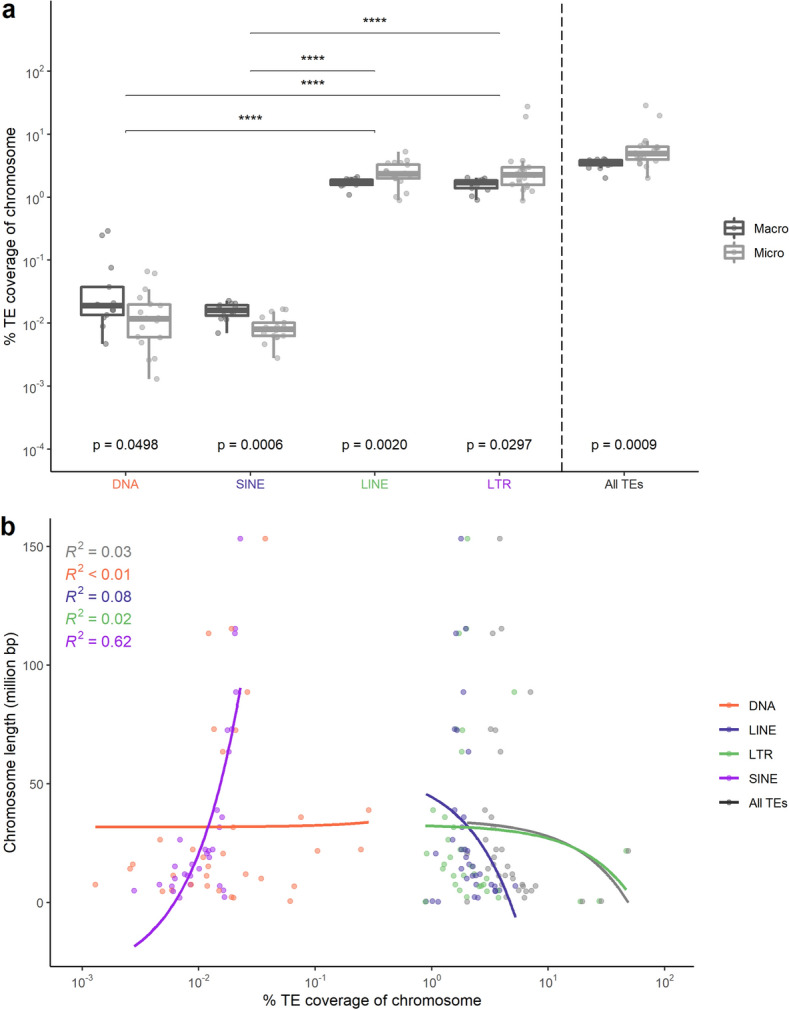
Relationships of the TEs and TE types to chromosomal characteristics. (**a**) Percentage of TE coverage for chromosomes separated based on macro and micro chromosomes. The coverage is presented for each TE type separately, and measures for all TEs together are shown to the left of the dotted line. P values are provided for Kruskal–Wallis tests comparing the means of micro and macro chromosomes (colour coded as in the legend) per type and for all TEs. Furthermore, significant comparisons were visualized with (*) (*p* ≤ 0.05 = *, *p* ≤ 0.01 = **, *p* ≤ 0.001 = *** and *p* ≤ 0.0001 = ****), of Kruskal–Wallis tests comparing the overall distributions of the different TE types and all TEs, all the significant p-values were < 2.22e-16. (**b**) Relative % TE coverage (log scale) of the chromosomes compared to chromosome length for all TEs (black) and per TE type separately (colour coded as in the legend). The only significant relationship between % relative TE coverage and chromosome length is observed for SINEs (*p* = 8.48e-8).

**Figure 3:**
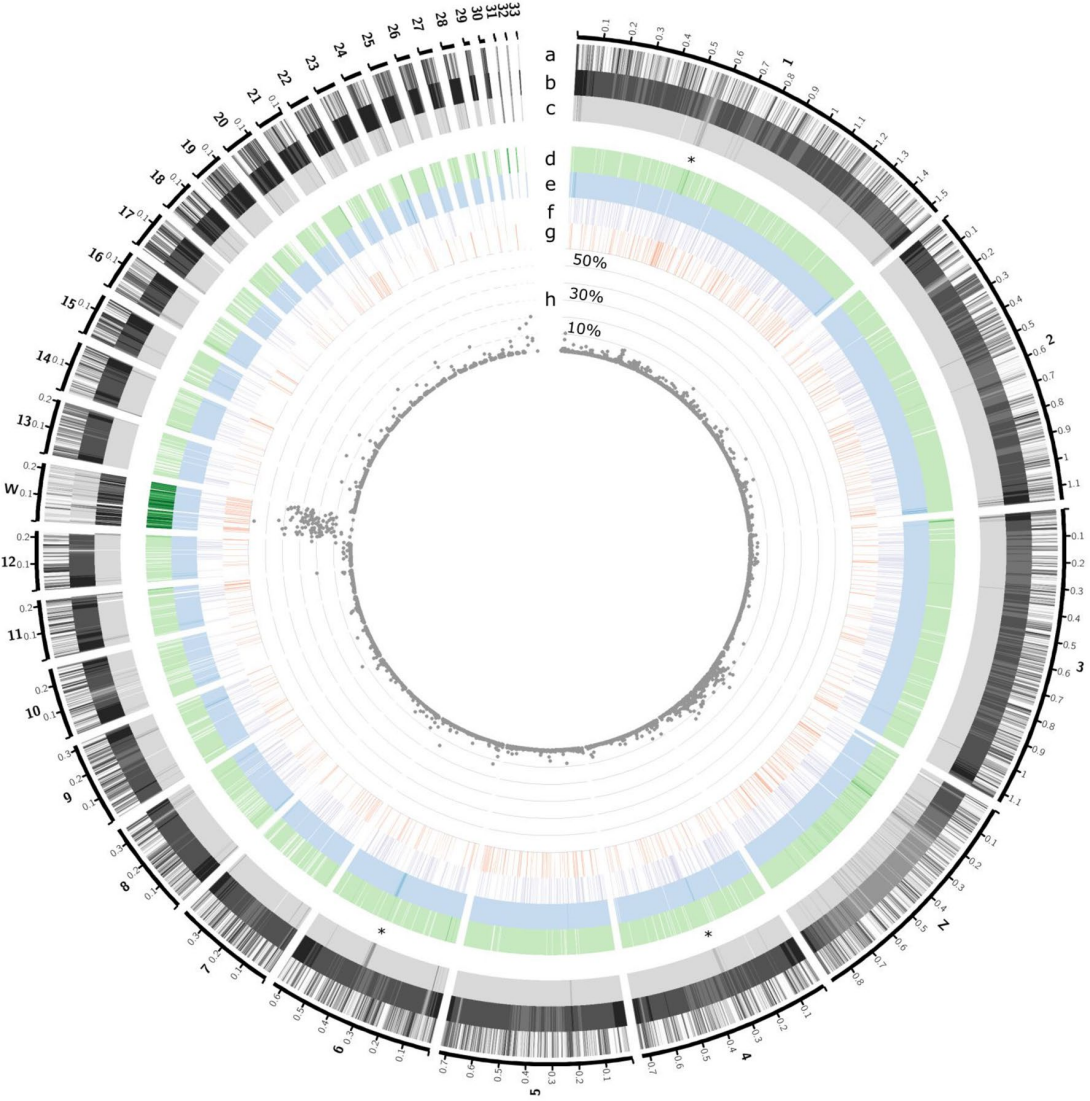
Genome-wide visualisation of detected TEs. All visualised features are calculated within windows of 200 kb. The respective chromosome number is indicated on the outside of the circos diagram. (**a**) Gene coverage (0 (white)–100 (black) %), (**b**) recombination rate (the higher the recombination rate, the darker), log 10 adjusted. TEs covered a 200 kb window to a maximum of 80%, in mainly the W chromosome (due to reduced recombination in sex chromosomes). To aid in visualising the lower registers of this distribution we narrowed our range for the TE tracks between 0 and 80% (from light to dark). Overall TEs and the TE types are colour-coded following the previous figures. In descending order (**c**) overall TE coverage, (**d**) LTR (green), (**e**) LINE (blue), (**f**) SINE (purple) and (**g**) DNA (red). The innermost track (h) shows the distribution of TE coverage (in 200 kb windows) of recent TEs, from 0 to 60% as this was the range occupied. The y-axis of this track illustrates the percentage of genome covered in increments of 10% (10, 30 and 50% labelled), while * highlight regions in autosomes with high levels of TE coverage.

Using an approach that in steps zooms deeper in on the genome we analysed the coverage of different types of TEs compared to chromosomal characteristics like chromosomal type (micro and macro chromosomes, with micro chromosomes defined as chromosomes with a length smaller than 20 Mb) and chromosome length. LTRs and LINEs tend to have a higher coverage across micro chromosomes compared to the macro chromosomes and vice versa for SINE and DNA transposons (Fig. 2a). Micro chromosomes tend to have a broader distribution of relative TE coverage, with two chromosomes having more than 10% TE coverage. When comparing the TE coverage of the chromosomes separated by TE type, they are significantly different between macro and micro chromosomes (Fig. 2a). No significant relationship between chromosome length and relative TE coverage is observed in global TE patterning, except for SINEs (*p* = 8.48e-8) (Fig. 2b). Within chromosomes, the different types of TEs are not uniformly distributed, showing high TE coverage regions in specific chromosomes (Fig. 3d-g, for example, chromosomes 1, 4 and 6, marked with *). Notably, the areas with high TE coverage tend to be located in different regions along the chromosome, and are dependent on the type of TE. In comparison to the autosomes, the sex chromosomes (Z and W) have overall elevated levels of TEs (mainly LTRs), with chromosome W for the majority of its length covered by TEs (Fig. 3c).

To further investigate the recent burst of TE activity (Fig. 1b), TEs were categorized into recent and ancient TE insertions based on their average Kimura-2 substitution level, with equal to and lower than 7 categorized as the recent TE insertions and anything above 7 as ancient TE insertions (for more information see materials and methods). The recent TE insertions cover 10,612,698 bp of the genome and therefore comprise 8,6% of the annotated TEs (through 9404 copies). The majority (93.2%) of these recent TE insertions belong to LTR retrotransposons. This results in 31.0% of the coverage assigned to LTRs being attributed to recent TE insertions and accounting for 19.4% of the total number of LTR copies. Within the genome, the majority of these recent TE insertions are located in the sex chromosomes, see Fig. 3h.

**Figure 4:**
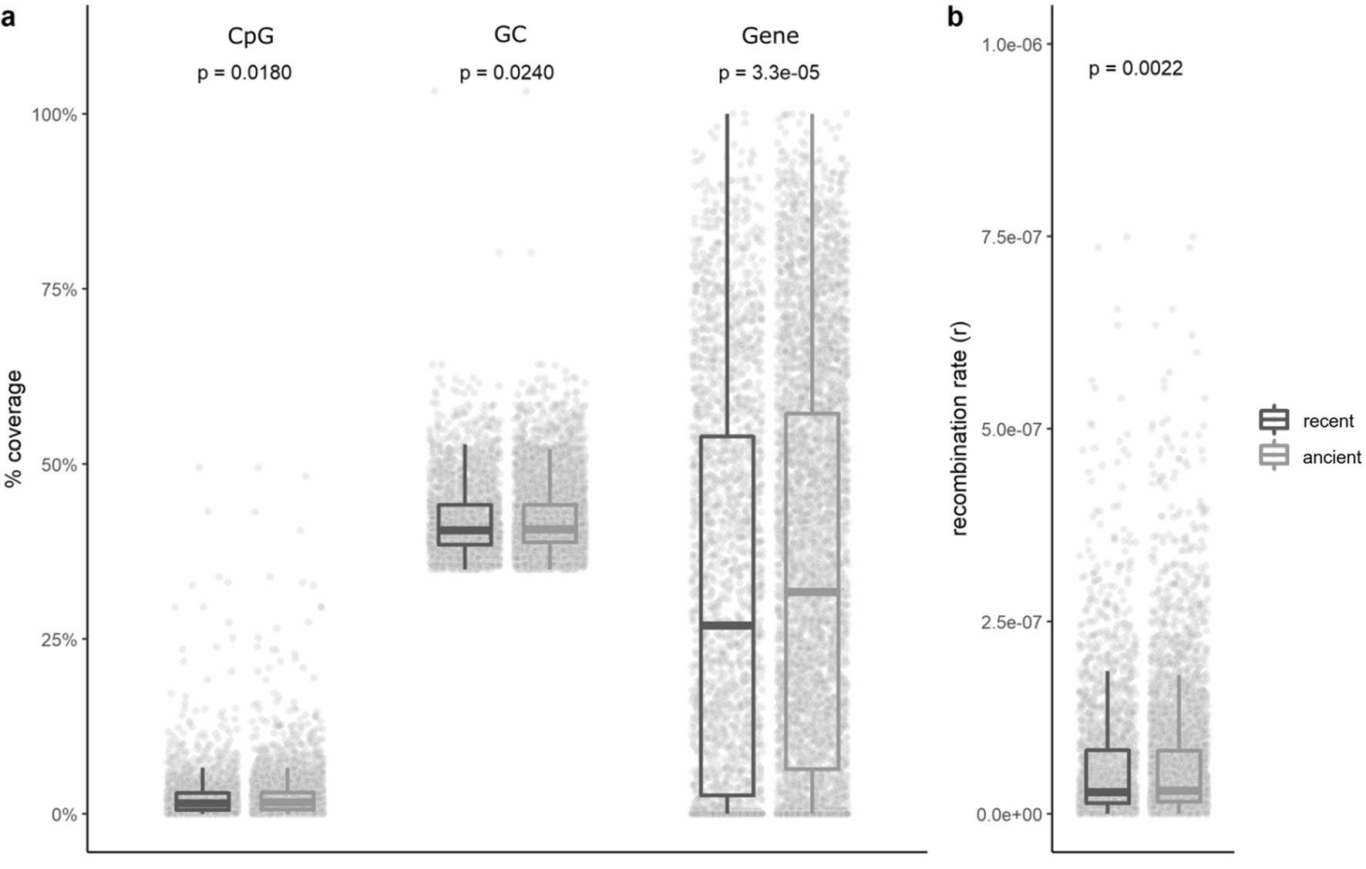
Relationships of recent and ancient TE insertions with the four genomic features studied. Recent and ancient colour coded as in ﻿legend. (**a**) From left to right distributions of percentage CpG island coverage, percentage GC coverage, and percentage gene coverage are shown, with (**b**) showing the distributions of recombination rate. For the comparison of the means of the distributions, the p-values (all significant) for the Kruskal–Wallis test performed are provided at the top of the figure.

As Fig. 3h shows that recent TE insertions are not uniformly distributed across the genome. We analysed how the coverage of recent TE insertions and ancient TE insertions are correlated to different genome features. Specifically, we focus on recombination rate, gene coverage, GC content and CpG island coverage all calculated in 200 kb windows (Table 2); recombination rate and gene coverage and their distributions along the genome are visualised in Fig. 3a,b. Partial Kendall’s rank correlation (partial r_τ_) was performed on all TEs, indicating a negative (but small) correlation of TE coverage with gene coverage and recombination rate (partial r_τ_: gene coverage: − 0.08, *p* = 8.6e-16 and recombination rate: − 0.12, *p* = 2.4e-40) and a slightly positive correlation with GC content (partial r_τ_: 0.08, *p* = 2.4e-19) (Table 2). Additionally, to account for the distinct influence of specific TE types (regardless of the age of the TE) we performed a similar analysis, revealing negative and significant correlations for LTRs, SINEs and DNA elements, while positive correlations are found for LINEs (Supplementary Table S1). Separate analyses with a particular focus on recent and ancient TE insertions reveal different relationships. As shown by the coverage distributions of recent and ancient TEs, they are significantly different for all four genomic features, Fig. 4a,b. By testing how the coverage of recent and ancient TE insertion categories correlate to the different features, the ancient TE insertions show a similar correlation pattern of GC content, recombination rate and gene coverage as was found for all TEs considered together, except for CpG island coverage which was slightly negatively correlated to TE coverage (partial r_τ_: − 0.03, *p* = 0.0068) (Table 2). Recent TE insertions were found to be negatively correlated to recombination rate (partial r_τ_: − 0.09, p = 1.4e-10), CpG island coverage (partial r_τ_: − 0.05, p = 0.0008) and gene coverage (partial r_τ_: − 0.08, * p* = 1.1e-09), while GC content was not correlated (partial r_τ_ non-significant) (Table 2). To account for the composition of TE type within the two categories we performed partial Kendall’s rank correlation per TE type coverage for each TE category, see Supplementary Table S1.

**Table 2:** Kendall’s rank correlation coefficients for different genome features and TE categories. Partial correlations were performed on (i) all TEs, (ii) ancient TE insertions and (iii) recent TE insertions. Significant values are highlighted in bold (*p* ≤ 0.05 = *, *p* ≤ 0.01 = **, *p* ≤ 0.001 = *** and *p* ≤ 0.0001 = ****), per genomic feature a Bonferroni correction was applied to account for multiple testing.

	GC	CpG	Gene	Rec.rate
All TEs	0.08****	0.01	− 0.08****	− 0.12****
Ancient	0.09****	− 0.03**	− 0.07****	− 0.02*
Recent	− 0.01	0.05***	− 0.08****	− 0.09****

Significant are in value [bold].

## Discussion

The overall TE composition for the Eurasian blackcap genome shows several typical characteristics found in other bird genomes. The average TE content in birds is 5–10%^2^, which is comparable to the 5.06% in the blackcap genome. Additionally, bird genomes typically show an abundance of LTR and LINE elements along with lower amounts of SINE and DNA elements^2^, similar to what was seen in our TE annotation. LINEs (mainly CR1 elements) were the most abundant non-LTR elements within our genome. Furthermore, the waves of activity seen within the blackcap for LINEs and LTRs are typical for birds^12^, however, the recent activity (kimura substitution level <  = 5) of LTRs that we see in the blackcap genome deviates from other bird genomes activity pattern. This deviation could result from a more complete annotation of recent TEs in the high-quality genome assembly available to us, in comparison to other (bird) species genome assemblies. Our annotation allowed the discovery of recent TE insertions covering 19.9% of TEs annotated, while accounting for only 8.6% of the copies found, meaning that complete TEs that were recently active and not mere fragments can be recovered fully. Here, we specifically report a recent burst of activity for ERVK LTR elements (Fig. 1 and Supplementary Fig. S1), with high levels of similarity to the original sequence. Whether these specific elements are currently active within the species, needs to be further investigated. Until higher quality genomes are assembled, and the diversity and activity of TEs within species are studied more extensively^5, 13^, it cannot be disentangled if the current activity of LTRs is present in other bird species as well or represents a deviation specific to the blackcap focally analysed here.

When evaluating the TE coverage on a chromosomal level we observe a non-uniform distribution along the chromosomes (Fig. 3c). In comparison to the autosomes, sex chromosomes possess a higher coverage of TEs, most prominently LTRs. This can easily be explained as a consequence of host purging mechanisms, like recombination, being almost absent in sex chromosomes^14, 15^. This is corroborated by the fact that we see a difference in the intra-chromosomal pattern for LTRs along the sex chromosomes compared to the autosomes (Fig. 3d). Furthermore, a large variation in TE coverage per chromosome is seen. This variability is mainly visible in micro chromosomes and is dependent on the type of TE evaluated (Fig. 2). Micro chromosomes arose through fission of macro chromosomes in the ancestral genome of birds, and have been found to support higher recombination rates, increased densities of genes, as well as GC content and CpG islands, compared to the macro chromosomes within the same genome^16, 17^. In the blackcap high occupation of LINEs and LTRs within micro chromosomes compared to the macro chromosomes, is seen, a particularly interesting observation, as previous research has instead found lower occupation of TEs in the micro chromosomes compared to the macro chromosomes in other bird species across the avian tree of life^16^. This finding is especially interesting as micro chromosomes are known to be highly conserved between remote bird species^17^. However, as discussed above, we currently lack TE annotations of more closely related species to the blackcap to determine if this is a blackcap specific deviation or more broadly found within birds.

As TEs are more acknowledged for their roles in trait evolution and speciation, investigating recent TE insertions becomes more important, to better understand their roles in evolution. While we do report recent TE insertions and their relation to different genomic features, the sustained activity of these TEs into current times needs to be further clarified. Looking at all TEs, across all autosomes we report similar relationships as in Bascón-Cardozo et al.^10^, see Table 2. Briefly, when taking the coverage of all TEs they are negatively correlated with recombination rate, gene coverage and positively correlated with GC content. These patterns are as expected, based on previous research^1, 3, 18^. However, when looking at the relationships of these genomic features towards the coverage of recent TE insertions the patterns change. These TEs are more likely to be in regions with lower levels of recombination rates and higher levels of CpG islands, as opposed to ancient TE insertions, see Table 2. These reported differences in relationships to the four genomic features can be explained by both the TE and the host genome. The TE landscape of the blackcap shows that recent TE insertions have been recently active (Fig. 1) as they have little accumulated base pair differences to the original sequence of the TE. As active TEs cause a threat to host genome stability, the host’s main defence is repression of TE activity through methylation, mediated by CpG islands^18^. This explains the slightly negative correlation found of ancient TE insertion coverage (Table 2), evidence of their evasion of the host’s defences. Our finding that the coverage of ancient TE insertions overall is slightly positively correlated with GC content can be explained by mutations accumulating within TEs, which naturally have higher levels of ATs^18^, resulting in host genome GC levels, over evolutionary time scales. Furthermore, we found a negative relationship between the coverage of recent TE insertions with recombination rate, contrasting with patterns of young LTR TEs previously found to positively associate with recombination rate in flycatchers^19^. However, our categorisation of recent TE insertions encompasses a wider age range of TEs, than can be considered “young”. It’s important to note that weighing in on these correlations is the TE type that composes the majority of a category, for example, the recent TE insertions are mainly comprised of LTRs, which were previously found to be negatively correlated to recombination rate^10^ and we also recover, see Supplementary Table S1. Interestingly, we do recover a positive correlation of coverage of recent LINE insertions and recombination rate. The differences we see based on TE type can potentially be attributed to the TE type specific method of inserting into the genome^1^, these type specific methods can result in insertion biases for the genomic regions in which they insert themselves. For example, LINEs occur more frequently in areas of the genome with increased recombination rate such as: promotors, genes and CpG islands, areas favourable as insertion site of LINEs^10^. To better reconstruct this relationship, further research focussing on recently active TEs and their placement near recombination hotspots is needed.

We provide a high-resolution characterisation of the TE landscape of the Eurasian blackcap, thereby aiding in the currently understudied field of TEs and their relation to genome features, with an emphasis on recent TE insertions. This TE annotation is not only a resource for future studies into TEs but can also aid in a better understanding of genomic variation within the blackcap and between different songbird species.

## Methods

The genome assembly was performed with the pipeline v1.5 of the Vertebrate Genomes Project (VGP) and can be found under NCBI BioProject PRJNA558064, accession number GCA_009819655.1, for further details on the sample collection and assembly see Ishigohoka et al.^9^. In brief, a female blackcap from mainland Spain was caught to extract genomic DNA. The following sequencing efforts went into the making of this reference genome: 80X Bionano optical maps, 60X PacBio long-read sequencing, 68X 10X-Genomics linked reads and 68X Arima HiC. Resulting in a high-quality genome with long contiguous stretches of DNA, with a chromosomal level resolution for 33 autosomal chromosomes and the sex chromosomes Z and W. To illustrate the high quality of our reference: the estimated genome size of the blackcap is 1.09 Gbp, and the reference N50 covers 7.06 Mbp.

### De novo TE prediction

Repetitive element consensus sequences were predicted de novo using RepeatModeler 1.0.11^20^. We additionally predicted the specific LTR class of TEs in the genome using LTRharvest^21^, with default settings, and the LTR-related hmm profiles from Pfam^22^ as input. LTRdigest^23^ was used to detect internal features of the LTR predictions, by running the LTRharvest output against the specific LTR protein domains (PFAM hmm profiles: PF07253, PF00077, PF08284, PF00078, PF07727, PF06817, PF06815, PF00075, PF00552, PF02022, PF00665, PF00098, PF00385, PF01393, PF00692, PF01021, PF03078, PF04094, PF08330, PF04195, PF05380). Candidate regions that did not include protein domains were removed. We independently, for each set of predicted sequences (RepeatModeler and LTRharvest) removed redundant sequences using usearch v7^24^ by clustering sequences by > 80% similarity.

All predicted sequences were searched against protein predictions of the gene annotation using diamond blastx 2.0.4^25^, we retained only (bitscore > 100) genes. Genes can sometimes be labelled as TEs and vice versa, as genes mislabelled as TEs will have one or two hits, while TEs often have multiple similar copies in the genome, and therefore will show multiple matching hits. Thus to confirm their identities, any potentially mislabelled gene and TE was submitted to eggnog^26^ for annotation. Any predicted TEs that could not be annotated were submitted to CENSOR^27^ to remove sequences with a score below < 200. The filtered RepeatModeler and LTRharvest annotations were then concatenated and merged into a single dataset using usearch v7 on 99% identity. All predicted repeats were renamed with the prefix: “Sylatr_”, the name of the repeat class and repeat family, using the renameRMDLconsensi.pl script^13^. The predicted library of consensus sequences is available in Supplementary Data 1.

### TE annotation

TEs were annotated in the genome, using the predicted library of consensus sequences, as well as two manually curated repeat libraries of the blue-capped cordon bleu^28^ and the collared flycatcher^13^ (most recent common ancestor to the blackcap estimated at 45.6 mya^29^), the repeat libraries were merged with 95% identity allowing the recovery of both species specific TEs as well as shared TEs between species. For this, RepeatMasker 4.1.0^30^ was run with the following parameters: -s-gccalc-a-x-poly-html-gff-u-xm-excln. Based on the results of the TE annotation, a TE landscape was created using the calcDivergenceFromAlign.pl and createRepeatLandscape.pl scripts as part of RepeatMasker 4.1.0 (See Fig. 1a). For each TE copy, the mutational distance to the consensus sequence was evaluated, to infer the Kimura 2-parameter distance. As the RepeatMasker output contains fragments of TEs, the Perl script “OneCodeToFindThemAll.pl” from Bailly-Bechet, Haudry, & Lerat^31^ was used to merge fragments into one TE copy. Using the “-strict” option we combined and filtered TEs based on the 80–80–80 rule^11^, resulting in the final TE annotation presented here, the gff file is available in the supplementary materials as Supplementary Data 2.

### Genome feature estimations

Genomic features, including gene density, recombination rate, GC content, and CpG islands were annotated in 200 kb windows as described in Bascón-Cardozo et al.^10^. Briefly, the gene annotation across the blackcap reference genome^9^ was generated with MAKER, using transposable element libraries from both, the collared flycatcher and blue-capped cordon-bleu (the gene annotation was conducted independently and before the construction of the TE library). A blackcap specific transcriptome was assembled from RNAseq and ISOseq data, to curate the predicted genes with high confidence^10^. Genes were also predicted from cDNA and protein sequences of three additional bird species, supporting accurate gene annotations. Furthermore, LD-based recombination rate estimation was performed using Pyrho^32, 33^, which estimates recombination rate (r) per base and generation using population-specific effective population sizes (Ne) and mutation rate and takes demography into account, unphased genotypes were inputted in VCF format, with optimized parameters for blackcaps as in Bascón-Cardozo et al.^10^. As mutation rate, we used estimates for the collared flycatcher, i.e. 4.6 × 10^–9^ site/generation^34^. Recombination rates were further calculated in non-overlapping windows taking account of the distance between pairs of sites for which recombination rates were available within each window. For both GC content and CpG islands, the calculations resulted in weighted averages per window.

### TEs relation with the blackcap genome

Accounting for different chromosome lengths, relative TE coverage was calculated (in %), for all TEs and separately per type, to understand the TEs distribution across the genome. We ran a linear regression to evaluate the relationship between TE coverage and chromosome length. Additionally, we tested the correlation of TE coverage between macro and micro chromosomes, with macro chromosome defined as > 20 Mb, for all TEs and per type, using a Kruskal–Wallis test.

### Recent TE insertions

We categorised TEs in recent and ancient categories by using the Kimura-2 substitution rate outputted by “OneCodeToFindThemAll” and using a threshold ≤ 7 for recent TE insertions. The threshold of ≤ 7 kimura-2 substitution level equates to a maximum age of ~ 16 million years ago (mya), based on the estimated mutation rate of the collared flycatcher at 2.3 × 10^–9^ mutations per site^13^. Basing our threshold on a tentative split from the blackcap’s most recent common ancestors to its sister species the garden warbler (*Sylvia borin*) at ~ 14–16 mya^29, 35^. As the split from the sister species has a wide margin, the decision was made to use the kimura-2 substitution distance corresponding to the latest split. By separating our TEs based on the split with the sister species we differentiate on whether the TEs were active before or after the species split. Different TE types have different lengths, which will affect the distinct categories. However, our categorisation into distinct age classes creates an additional characteristic for each TE category. Specifically, this concerns the level of fragmentation, with ancient TE insertions being more fragmented than recent TE insertions. To not count TE fragments of one TE insertion multiple times (and thereby inflating the number of TEs), we quantify the genomic occupation of TEs as the percentage coverage of base pairs by TEs in a genomic window. For both categories, TE coverage was calculated in 200 kb windows. Focusing our analysis on the autosomes, we tested the distribution of the two categories of TEs to the genomic features with a Kruskal–Wallis test. We wanted to know how the relationships differed for recent TE insertions and ancient TE insertions, in both the repeat landscape and the relations of TEs with genomic features, such as recombination rate, GC content, CpG islands and gene coverage which had been seen to correlate in blackcaps^10^. To allow for a direct comparison with Bascón-Cardozo et al.^10^, partial Kendall’s rank correlation test was initially performed on all TEs and the different TE types. Additionally, we tested the correlation of all genomic features within the two categories of TEs separately, using a partial Kendall’s rank correlation test. We decided on the partial Kendall’s rank correlation test to account for the high correlation between the genomic features. Additionally, we used a Bonferroni correction on the p-values to account for multiple testing, this was done either for the different categories (Table 2) or along the different categories and TE types (supplementary Table S1).

Statistical tests were performed using R^36^, package ppcor^37^, and visualised using ggplot2 and ggpmisc^38, 39^, as well as circos, to display genome variation in circos plots^40^.

### Supplementary Information

Supplementary Information 1.

Supplementary Information 2.

Supplementary Information 3.

## Data Availability

The reference genome of the European blackcap can be found under NCBI BioProject PRJNA558064, accession numbers GCA_009819655.1 and GCA_009819715.1 (Ishigohoka et al. 2021). Gene annotation is deposited at Zenodo (https://zenodo.org/deposit/7813728#). The TE consensus sequences and gff are provided along with this submission as supplementary materials. Additional data is deposited to GitHub (https://github.com/Karenbc/Recombination-rates-and-genomic-features-Blackcap) as part of Bascón-Cardozo et al. 2022.
