# Supplementary material for: The blackcap (Sylvia atricapilla) genome reveals a recent accumulation of LTR retrotransposons
Source: Sci Rep. 2023 Sep 30;13:16471. doi: 10.1038/s41598-023-43090-1 (PMC10542752; doi:10.1038/s41598-023-43090-1)
Supplement: Supplementary file 1 — Supplementary Information 1. [file 41598_2023_43090_MOESM1_ESM.pdf]

The blackcap (*Sylvia atricapilla*) genome reveals a recent accumulation of LTR retrotransposons

Andrea Bours[1*], Peter Pruisscher[1,2], Karen Bascón-Cardozo[1], Linda Odenthal-Hesse[3], Miriam Liedvogel[1,4*]

Andrea Bours and Peter Pruisscher should be considered as joint first authors.

* Corresponding authors

[1] MPRG Behavioural Genomics, Max Planck Institute for Evolutionary Biology, 24306 Plön, Germany

[2] Department of Evolutionary Biology, Evolutionary Biology Centre (EBC), Uppsala University, Sweden

[3] Department Evolutionary Genetics, Max Planck Institute for Evolutionary Biology, 24306 Plön, Germany

[4] Institute of Avian Research 'Vogelwarte Wilhelmshaven', 26386 Wilhelmshaven, Germany

SUPPLEMENTARY INFORMATION

Supplementary Tables:

Supplementary Table S1: Partial Kendall's rank correlation per TE type. Partial correlations were performed per TE type, and for each category. Significant values are highlighted in bold, and levels of significance were visualized with (*) (p≤ 0.05 = *, p≤ 0.01 = **, p≤ 0.001 = *** and p≤ 0.0001 = ****). To account for multiple testing a Bonferroni correction was applied, per genomic feature.

	**TE category**	**GC**	**CpG.cov**	**Gene.cov**	**Rec.rate**
LTR	all	0.01	-0.01	**-0.10********	**-0.14********
	ancient	-0.005	**-0.04******	**-0.09********	**-0.04********
	recent	0.04	-0.01	**-0.11********	**-0.07********
LINE	all	**0.08********	-0.01	-0.01	-0.01
	ancient	**0.10********	-0.01	-0.02	0.01
	recent	**0.14********	-0.02	-0.02	**0.05****
SINE	all	**-0.10********	-0.002	0.02	**-0.06********
	ancient	**-0.09********	0.00001	0.01	**-0.07********
	recent	**-0.11********	0.0001	0.01	**-0.07********
DNA	all	0.01	0.004	**-0.03****	**-0.10********
	ancient	0.003	-0.01	**-0.03***	**-0.06********
	recent	0.03	-0.01	-0.03	**-0.10********

Supplementary figures:

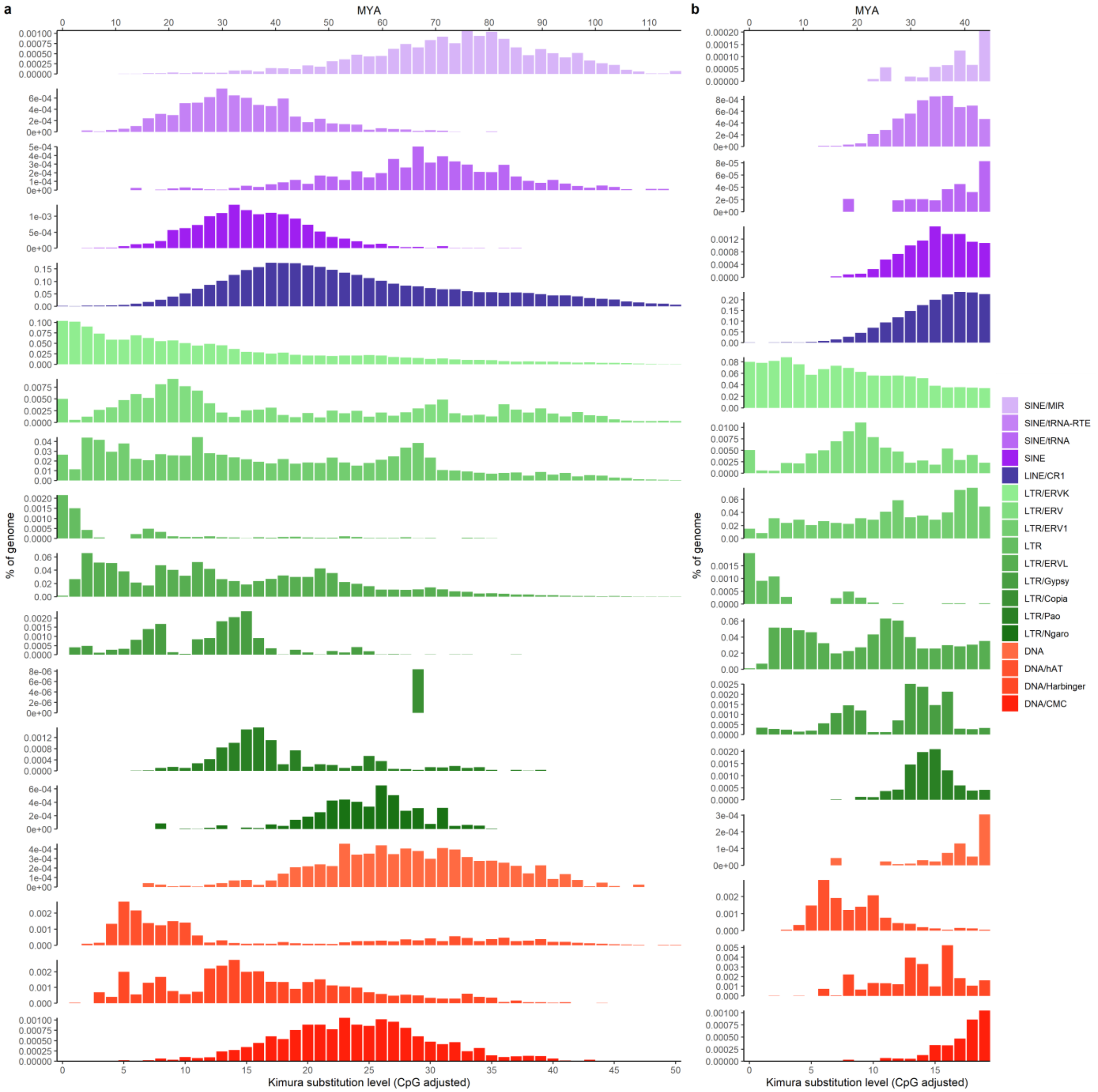

Supplementary Figure S1: interspersed repeat landscape of the blackcap genome separately plotted per family. Plots not to scale on the Y axis to visualise distribution patterns of repeat families with generally lower % coverage. a) Repeat landscape calculated by RepeatMasker on the raw output. b) Repeat landscape calculated on the final TE annotation. The bottom x-axis is the kimura-2 substitution level, the top x-axis is the timescale in million years ago (MYA), and the y-axis is the percentage of the genome occupied.
